# Dataset of *Centella Asiatica* leaves for quality assessment and machine learning applications

**DOI:** 10.1016/j.dib.2024.111150

**Published:** 2024-11-23

**Authors:** Rohini Jadhav, Mayuri Molawade, Amol Bhosle, Yogesh Suryawanshi, Kailas Patil, Prawit Chumchu

**Affiliations:** a Vishwakarma University, Pune, India; b Kasetsart University, Sriracha, Thailand; c Bharati Vidyapeeth (Deemed to be University) College of Engineering, Pune, India; d MIT Art, Design and Technology University, Pune, India

**Keywords:** Artificial intelligence, Brahmi, Medicinal plant, Quality assessment, Machine learning

## Abstract

*Centella asiatica* is a significant medicinal herb extensively used in traditional oriental medicine and gaining global popularity. The primary constituents of *Centella asiatica* leaves are triterpenoid saponins, which are predominantly believed to be responsible for its therapeutic properties. Ensuring the use of high-quality leaves in herbal medicine preparation is crucial across all medicinal practices. To address this quality control issue using machine learning applications, we have developed an image dataset of *Centella asiatica* leaves. The images were captured using Samsung Galaxy M21 mobile phones and depict the leaves in “Dried,” “Healthy,” and “Unhealthy” states. These states are further divided into “Single” and “Multiple” leaves categories, with “Single” leaves being further classified into “Front” and “Back” views to facilitate a comprehensive study. The images were pre-processed and standardized to 1024 × 768 dimensions, resulting in a dataset comprising a total of 9094 images. This dataset is instrumental in the development and evaluation of image recognition algorithms, serving as a foundational resource for computer vision research. Moreover, it provides a valuable platform for testing and validating algorithms in areas such as image categorization and object detection. For researchers exploring the medicinal potential of *Centella asiatica* in traditional medicine, this dataset offers critical information on the plantʼs health, thereby advancing research in herbal medicine and ethnopharmacology.

Specifications Table

**Table:** 

Subject	Applied Machine Learning, Agriculture
Specific subject area	Agronomy & Crop Science
Data format	Raw
Type of data	Image
Data collection	The leaves from 50 plants of *Centella asiatica* were collected from the Vishwakarma University campus in Pune. Three types of leaf conditions were considered for image creation: Dried, Healthy, and Unhealthy. Total 9094 high-resolution images captured using a Samsung Galaxy M21 Android phone with a 12-megapixel sensor, 4.6 mm focal length, F2 aperture, and 4000 × 3000 resolution, featuring leaf images taken under different backgrounds, angles, and lighting conditions. These images were divided into “Single” and “Multiple” leaf categories, with “Single” leaves being further classified into "Front" and “Back” views to enable a comprehensive study. The images were pre-processed and standardized to 1024 × 768 dimensions, resulting in a dataset comprising a total of 9094 images in 1024 × 768 dimensions. The completed dataset was uploaded to Mendeley Data.
Data source location	Vishwakarma University, Kondhwa Budruk, Maharashtra, Pune, India Latitude: 18.4605° N Longitude: 73.8837° E
Data accessibility	Repository name: Centella Asiatica Leaf Image Dataset Data identification number: 10.17632/hrx2kgphy5.2 Direct URL to data: https://data.mendeley.com/datasets/hrx2kgphy5/2

## 1 Value of the Data

• This dataset represents the first documented compilation of high-resolution images specifically capturing *Centella asiatica* leaves, an herb celebrated in Ayurvedic medicine for cognitive enhancement and frequently used as a substitute for *Bacopa monnieri*. By offering a standardized image collection, this dataset addresses the need to distinguish *Centella asiatica* from similar medicinal plants, providing a valuable resource for future machine learning models aimed at detecting adulteration.
• Comprising 9094 captured images of *Centella asiatica* leaves in diverse health conditions (healthy, unhealthy, and dried), this dataset allows for in-depth exploration of plant health status. Such variation in leaf conditions offers machine learning researchers a robust dataset that supports accurate categorization and analysis, with potential applications in real-time agricultural monitoring.
• The images have been carefully preprocessed and uniformly scaled to 1024 × 768 pixels, enhancing consistency across the dataset. This preprocessing not only supports various machine learning applications but also ensures the dataset's suitability for developing scalable image recognition models, paving the way for advancements in plant classification and health assessment.
• This dataset offers opportunities for computer vision researchers to explore innovative approaches in pattern recognition and enhance classification techniques for medicinal plant species.

## 2 Background

*Bacopa monnieri*, also known as Brahmi, is renowned for its benefits in memory improvement, treatment of insomnia and epilepsy, and its anxiolytic properties. Similarly, *Centella asiatica* is used to heal wounds, enhance mental clarity, and treat skin conditions such as leprosy and psoriasis. Both plants possess unique medicinal properties and are highly valued in traditional medicine. Due to demand and supply issues, *Centella asiatica* is sometimes sold as Brahmi in various products. This has led to the use of both plant leaves interchangeably to meet market demands, resulting in adulteration. To address this problem and accurately identify *Centella asiatica* leaves, we decided to create a comprehensive image dataset of *Centella asiatica* leaves. This dataset will help in identifying *Centella asiatica* plant leaves using machine learning applications, thereby ensuring the integrity of herbal products [[1], [2], [3], [4], [5]].

## 3 Data Description

*Centella asiatica*, widely known by names such as Indian pennywort, Asiatic pennywort, spadeleaf, coinwort, gotu kola, and Mandukaparni, is a herbaceous, perennial plant in the Apiaceae family. Revered in Ayurvedic medicine, this plant is recognized for its cognitive-enhancing properties and is also employed in wound healing and treating skin conditions like psoriasis [6]. To support precise identification and quality assessment of *Centella asiatica* leaves, we have curated an extensive, high-resolution leaf image dataset. This dataset is designed to meet the specific needs of machine learning applications focused on plant identification, classification, adulteration detection, and health status evaluation, making it a valuable tool for both agricultural and medicinal research.

The *Centella asiatica* dataset represents a unique and targeted contribution, standing apart from existing leaf image datasets in multiple key aspects. Unlike general plant or herb datasets, it is among the first to focus specifically on *Centella asiatica*—an herb of significant medicinal importance, renowned for cognitive-enhancing and wound-healing properties. While existing other datasets, like those for papaya, fig, mint, groundnut, sugarcane, lemongrass leaves, provide useful insights into plant health, they lack the medicinal and pharmacological relevance that *Centella asiatica* holds [[7], [8], [9], [10], [11], [12]]. For instance, the papaya dataset [8] includes diseased and healthy samples but is limited to five categories and around 1400 images, whereas our dataset offers a larger sample size of 9094 images with detailed categories, including single and multiple leaf orientations and distinct front and back views. This nuanced categorization enables machine learning applications, such as detecting adulteration with similar herbs like *Bacopa monnieri*. Additionally, the dataset's high-quality resolution (1024 × 768 pixels) enhances compatibility with machine learning models, aiding in fine-grained image analysis for plant health, quality assessment, and digital herbarium development.

This *Centella asiatica* dataset is structured into three main folders representing various states of leaf health: Dried (2996 images), Healthy (3048 images), and Unhealthy (3050 images). This organization enables machine learning models to classify leaves based on health status, facilitating plant quality assessment and real-time health monitoring in agricultural systems. Each main folder is further divided to capture detailed leaf views, thereby supporting complex image recognition and classification tasks.

• *Healthy* Folder: This folder includes 3048 images and is further divided into Single (2044 images) and Multiple (1004 images) subfolders. The Single subfolder contains images of individual leaves and is further categorized by leaf orientation into Front (1014 images) and Back (1030 images) views, representing the dorsal and ventral sides. These distinctions allow models to capture subtle differences in leaf structure and texture, crucial for accurate plant identification and quality assessment.
• *Unhealthy* Folder: Comprising 3050 images, this folder is similarly organized into Single (2024 images) and Multiple (1016 images) subfolders. The Single images are further divided into Front (1023 images) and Back (1011 images) views, which capture visible indicators of plant health degradation. This detailed categorization enhances the dataset's applicability in machine learning tasks focused on early disease detection and visual health assessment, contributing to improved agricultural management practices.
• *Dried* Folder: This folder contains 2996 images of dried leaves, which are organized into Single (2018 images) and Multiple (985 images) subfolders. Unlike the healthy and unhealthy categories, the Single images of dried leaves are not divided into front and back views due to the shriveled nature of dried leaves, which obscures these distinctions. Including dried leaves offers additional insights into post-harvest conditions and storage impacts, supporting studies on leaf preservation and medicinal quality retention.

This comprehensive dataset not only facilitates *Centella asiatica* identification but also supports a range of machine learning applications in plant health assessment, quality control, and agricultural decision-making. It is poised to aid researchers in developing machine learning models for the detection of adulteration in herbal products, a critical need in ensuring the authenticity of herbal medicine. Additionally, the dataset's structured diversity in health states and orientations makes it an invaluable resource for computer vision researchers focused on fine-grained plant image classification and the development of robust, application-ready models. By offering categorized, high-quality images, this dataset provides a foundation for advancing both applied research and the broader fields of ethnobotany and agricultural informatics.

**Fig. 1:**
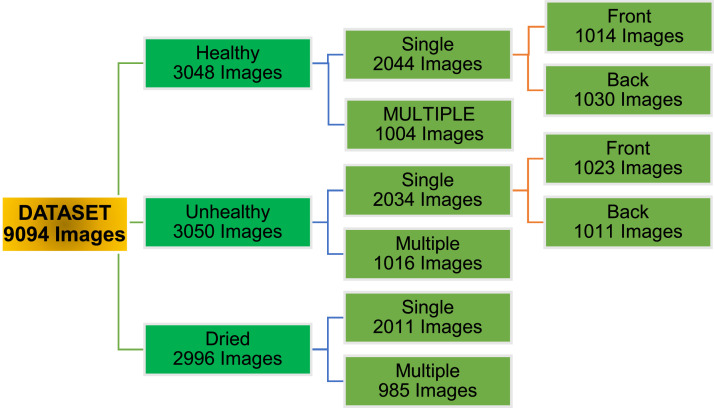
Directory structure of the dataset.

Fig. 1. illustrates the directory structure of the *Centella asiatica* plant leaf dataset, while Table 1 displays sample images from the dataset.

**Table 1:** Sample images of *Centella asiatica* leaves dataset.

## 4 Experimental Design, Materials and Methods

### 4.1 Experimental setup

Leaves from *Centella asiatica* 50 plants were gathered from the herbal garden located at Vishwakarma University Pune (coordinates: 18°27′34.8″N 73°53′01.1″E) between January 2024 and March 2024. A total of 50 individual plants were specifically chosen for leaf collection to compile the dataset. Approximately 400 individual leaves were selected for each category (Healthy, Unhealthy, Dried). Healthy leaves with a moisture content below 5 % were classified as ‘dried’, with moisture levels determined using the oven-drying method [13]. Leaves of *Centella asiatica* exhibiting any visual discoloration, aside from the green hue, were categorized as unhealthy. Conversely, leaves that were uniformly green, without any discoloration, were classified as healthy. The distinction between healthy and unhealthy leaves was based solely on visual appearance.

**Fig. 2:**
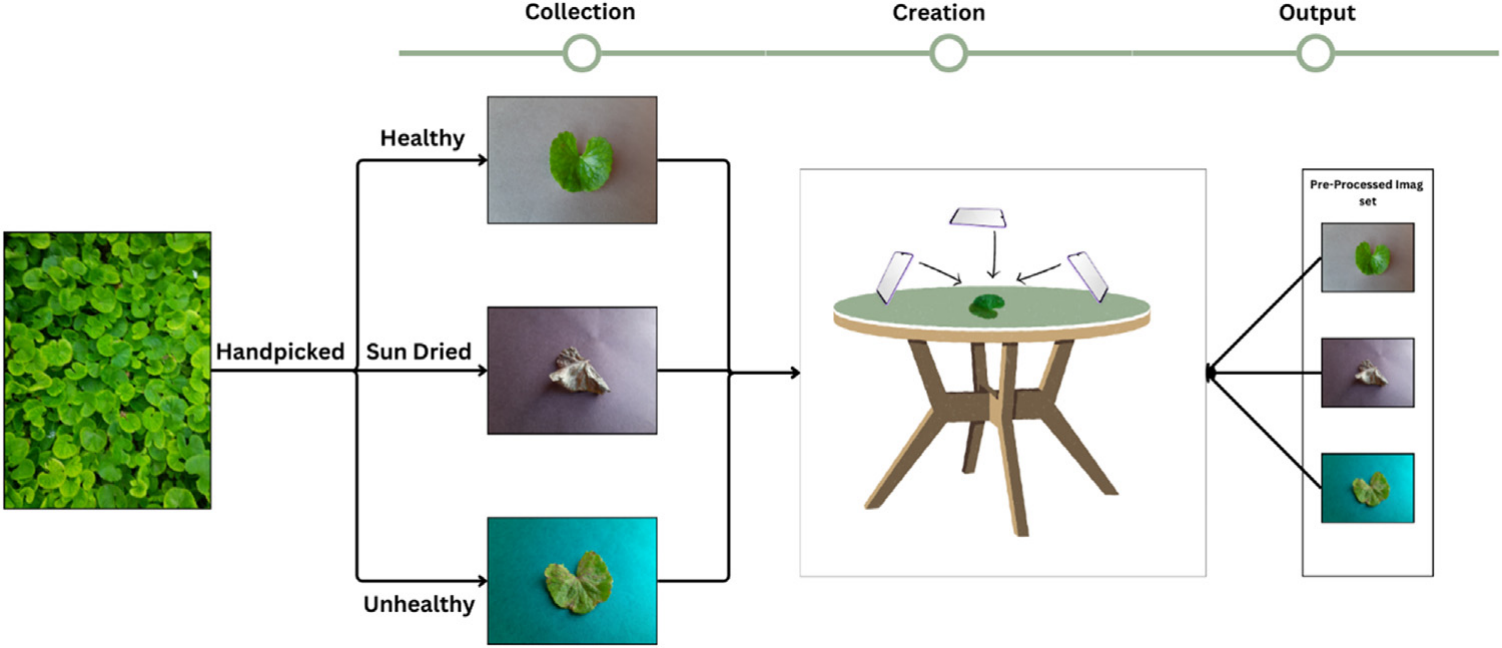
Experimental Setup for *Centella asiatica* leaves dataset.

Images of leaves from each selected plant were taken under varying backgrounds, angles, and lighting conditions. The experimental setup for creating the *Centella asiatica* leaf image dataset is depicted in Fig. 2.

### 4.2 Camera specifications

The dataset comprises a total of 9094 high-resolution images captured utilizing a Samsung Galaxy M21 Android phone equipped with a 12 Mega pixel sensor, a focal length of 4.6 mm, an aperture range of F2, and a resolution of 4000 × 3000.

### 4.3 Image pre-processing

**Fig. 3:**
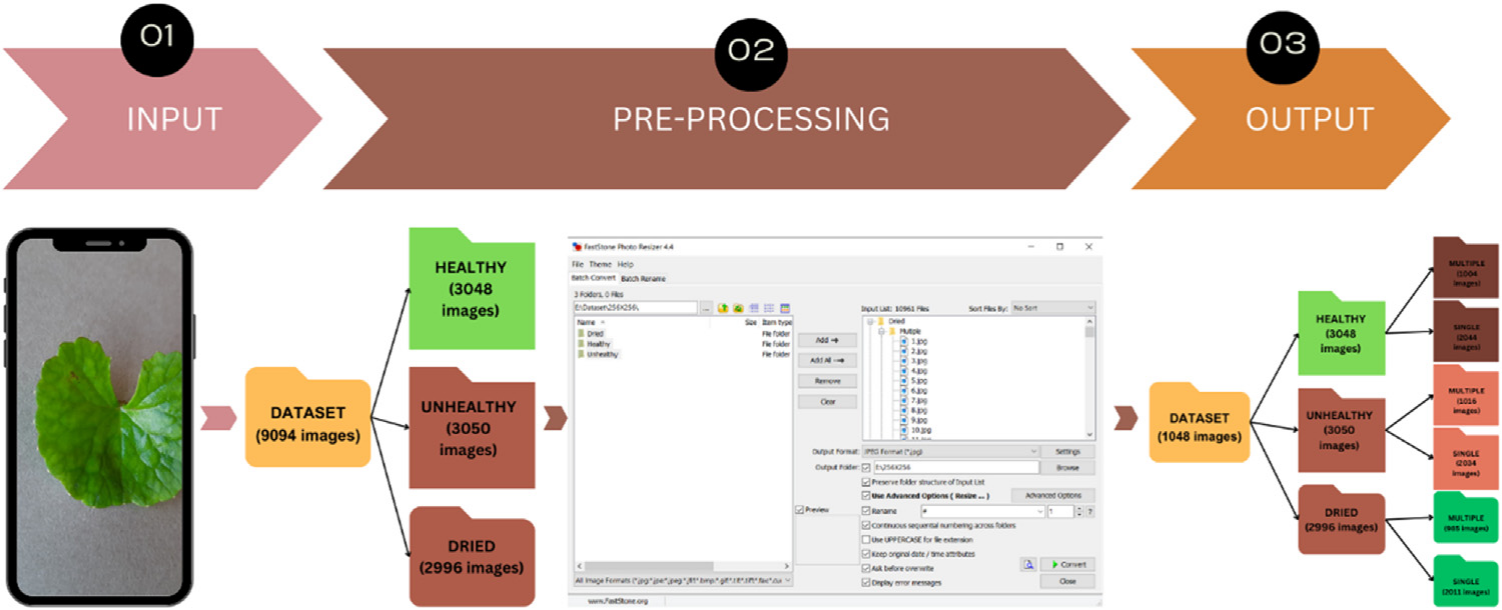
Stepwise preprocessing process.

To ensure image uniformity, pre-processing was conducted using FastStone Photo Resizer software. This tool was employed to systematically adjust the dimensions of the input images according to specified resolution parameters [14]. This process standardized the resolution across all images in the dataset, promoting consistency and uniformity. Such pre-processing is crucial in data preparation as it enhances image quality and ensures compatibility for subsequent analyses and applications. We reduced the image resolution from 4000 × 3000 pixels to 1024 × 768 pixels to enhance computational efficiency, reduce model training time, and ensure consistency for machine learning applications. The resultant images were saved in JPG format and resized to a resolution of 1024 × 768 pixels, with all files renamed using numerical identifiers only (Fig. 3). The dataset was uploaded to Mendeley Data, where it is now publicly accessible [15].

## Limitations

The dataset is collected from a specific region, potentially limiting its applicability to other geographical areas with different disease prevalence or manifestations.

## Ethics Statement

Our study does not involve studies with animals or humans. Therefore, we confirm that our research strictly adheres to the guidelines for authors provided by Data in terms of ethical considerations.

## Credit Author Statement

**Rohini Jadhav:** Data Curation; Methodology; **Mayuri Molawade:** Methodology; Software **Amol Dhumane:** Formal analysis, Validation; **Yogesh Suryawanshi:** Conceptualization, Visualization, Writing– original draft **Kailas Patil:** Supervision, Writing – original draft; **Prawit Chumchu:** Writing – review & editing.

## Data Availability

Mendeley DataCentella Asiatica Leaf Image Dataset (Original data).

## References

[1] Yadav A., Ahmad J., Chaudhary A.A., Ahmad A. (2012). Development of sequence characterized amplified region (SCAR) marker for the authentication of Bacopa monnieri (L.) Wettst. Eur. J. Med. Plants.

[2] Shah A.P., Travadi T., Sharma S., Pandit R., Joshi C., Joshi M. (2022). Comprehensive analysis using DNA metabarcoding, PCR, and HPLC unveils the adulteration in Brahmi herbal products. bioRxiv.

[3] Jadhav R., Suryawanshi Y., Bedmutha Y., Patil K., Chumchu P. (2023). Mint leaves: dried, fresh, and spoiled dataset for condition analysis and machine learning applications. Data Br..

[4] Thite S., Suryawanshi Y., Patil K., Chumchu P. (2023). Coconut (Cocos nucifera) tree disease dataset: a dataset for disease detection and classification for machine learning applications. Data Br..

[5] Sun X., Qian H. (2016). Chinese herbal medicine image recognition and retrieval by convolutional neural network. Plos One.

[6] Gohil K.J., Patel J.A., Gajjar A.K. (2010). Pharmacological review on Centella asiatica: a potential herbal cure-all. Indian J. Pharm. Sci..

[7] Aishwarya M.P., Reddy A.P. (2023). Dataset of groundnut plant leaf images for classification and detection. Data Br..

[8] Gani R., Rashid M.R.A., Ahmed J., Isty M.N., Islam M., Hasan M., Ali M.S. (2024). Smartphone image dataset to distinguish healthy and unhealthy leaves in Papaya Orchards in Bangladesh. Data Br.

[9] Choompookham T., Okafor E., Surinta O. (2024). Mulberry leaf dataset for image classification task. Data Br..

[10] Hafi S.J., Mohammed M.A., Abd D.H., Alaskar H., Alharbe N.R., Ansari S., Hussain A.J. (2024). Image dataset of healthy and infected fig leaves with Ficus leaf worm. Data Br..

[11] Patil K., Suryawanshi Y., Patrawala A., Chumchu P. (2024). A comprehensive lemongrass (Cymbopogon citratus) leaf dataset for agricultural research and disease prevention. Data Br..

[12] Thite S., Suryawanshi Y., Patil K., Chumchu P. (2024). Sugarcane leaf dataset: a dataset for disease detection and classification for machine learning applications. Data Br..

[13] Sadasivam S. (1996).

[14] Patil K., Suryawanshi Y., Dhoka A., Chumchu P. (2024). Plumbago Zeylanica (Chitrak) leaf image dataset: a comprehensive collection for botanical studies, herbal medicine research, and environmental analyses. Data Br..

[15] Suryawanshi Y., Wakode K., Patil K., Prawit C. (2024). Centella asiatica leaf image dataset. Mendeley Data.

